# New Parameters to Quantitatively Express the Invasiveness of Bacterial Strains from Implant-Related Orthopaedic Infections into Osteoblast Cells

**DOI:** 10.3390/ma11040550

**Published:** 2018-04-03

**Authors:** Davide Campoccia, Lucio Montanaro, Stefano Ravaioli, Ilaria Cangini, Francesca Testoni, Livia Visai, Carla Renata Arciola

**Affiliations:** 1 Research Unit on Implant Infections, Rizzoli Orthopaedic Institute, 40136 Bologna, Italy; davide.campoccia@ior.it (D.C.); lucio.montanaro@unibo.it (L.M.); stefano.ravaioli@ior.it (S.R.); ilaria.cangini@irst.emr.it (I.C.); francesca.testoni@ior.it (F.T.); 2 Department of Experimental, Diagnostic and Specialty Medicine, University of Bologna, 40126 Bologna, Italy; 3 Department of Molecular Medicine, Center for Tissue Engineering (CIT), INSTM UdR of Pavia, University of Pavia, 27100 Pavia, Italy; livia.visai@unipv.it; 4 Department of Occupational Medicine, Ergonomy and Disability, Nanotechnology Laboratory, Salvatore Maugeri Foundation, 27100 Pavia, Italy

**Keywords:** orthopaedic implant infections, bacterial internalization, *staphylococcus aureus*, *staphylococcus epidermidis*, *staphylcoccus lugdunensis*, *enterococcus faecalis*, osteoblasts

## Abstract

Complete eradication of bacterial infections is often a challenging task, especially in presence of prosthetic devices. Invasion of non-phagocytic host cells appears to be a critical mechanism of microbial persistence in host tissues. Hidden within host cells, bacteria elude host defences and antibiotic treatments that are intracellularly inactive. The intracellular invasiveness of bacteria is generally measured by conventional gentamicin protection assays. The efficiency of invasion, however, markedly differs across bacterial species and adjustments to the titre of the microbial inocula used in the assays are often needed to enumerate intracellular bacteria. Such changes affect the standardisation of the method and hamper a direct comparison of bacteria on a same scale. This study aims at investigating the precise relation between inoculum, in terms of multiplicity of infection (MOI), and internalised bacteria. The investigation included nine *Staphylococcus aureus*, seven *Staphylococcus epidermidis*, five *Staphylococcus lugdunensis* and two *Enterococcus faecalis* clinical strains, which are co-cultured with MG63 human osteoblasts. Unprecedented insights are offered on the relations existing between MOI, number of internalised bacteria and per cent of internalised bacteria. New parameters are identified that are of potential use for qualifying the efficiency of internalization and compare the behaviour of bacterial strains.

## 1. Introduction

Internalization in non-professional phagocytes has been reported to be an important pathogenetic mechanism actuated by bacteria to elude host defences [1] and medical therapies [2]. Following intracellular invasion of host cells, bacteria can actually hide from immune defences as well as medical therapies that are based on conventional antibiotics such as aminoglycosides, which are not intracellularly active. Once internalised, bacteria have been found to face distinct fates: they can either multiply and lyse the host cells or, alternatively, become dormant and transiently persist within the cytoplasm [3]. In the latter case, invaded host cells represent persistence niches potentially responsible for the relapse of infection at the end of medical treatments [3]. 

Bacterial persistence and occurrence of relapse bear relevant implications, especially in infections associated to biomaterial implants. These infections are difficult to treat and often result in septic failure of the implant with need for implant replacement. The difficult eradication of bacteria persisting in the tissues determines a high risk of reinfection even for the new implant. 

The possibility of intracellularly invading eukaryotic cells is well established for a variety of pathogenic species across different genera, including: *Mycobacterium*, *Escherichia*, *Salmonella*, *Listeria*, *Shigella*, *Legionella* (e.g., *L. pneumophila*), *Chlamydia* (e.g., *C. pneumoniae*), *Yersinia* (e.g., *Y. pestis* and *Y. pseudotuberculosis*), *Streptococcus* (e.g., groups A, B, C, and G; *S. viridans*; and *S. pneumoniae*) and others [4]. Implant related infections are prevalently caused by *Staphylococcus aureus*, coagulase-negative staphylococci (CNS) (*Staphylococcus epidermidis*, in first place), and by several other Gram-negative and Gram-positive opportunistic species, including *Enterococcus faecalis*. While numerous studies have well documented the special ability of *S. aureus* to adhere to and internalise into eukaryotic cells, there is still a lack of information on the properties of cell invasiveness for many other opportunistic species. In recent years, raising attention has become focussed on these less investigated species whose cellular invasiveness has not been fully explored. 

Together with the work of the Lyon BJI Study Group [5], our past investigations have contributed to clarify that bone cells invasion is not a clinically relevant pathogenetic mechanism among clinical isolates of opportunistic species such as *S. epidermidis* and *S. lugdunensis* [4]. The same consideration applies to orthopaedic clinical isolates of *E. faecalis* [4]. Recently, after a comprehensive screening of different staphylococcal species, Maali et al. (2016) reported that *S. pseudintermedius* was actually the only staphylococcal species, apart from *S. aureus*, able to consistently invade osteoblast cells [6].

In our past studies, we were able to reveal some neat differences of internalization when comparing different species to *S. aureus*. However, our work was based on the state of the art of previous scientific literature. Accordingly, different inoculum titres had to be adopted depending on the bacterial species tested. A multiplicity of infection (MOI) of 100:1, i.e., 100 viable bacteria per osteoblast cell, was the inoculum titre used for testing *S. aureus*. The inoculum size increased to 500:1 for *S. epidermidis* [5,7] and reached 1000:1 for *S. lugdunensis* (whose internalization had previously been tested just by an assay based on Fluorescence-activated cell sorting technique [8]) and *E. faecalis* [9]. These data, showing rapid and efficient internalization at a low inoculum size for *S. aureus* and, on the other hand, inefficient internalization at a high inoculum for the other species, were of clear interpretation. However, this was certainly not an ideal system to analyse and compare internalization of bacteria across and within species on the same ground. Such conventional approach, critically depending on the inoculum size, can be thought appropriate for rapid screenings, but with some limitations for a fine standardised evaluation of the efficiency of invasiveness across different species/strains, given the diverse testing conditions. Moreover, in this type of studies, the colony forming units (CFU) titre of the bacterial inoculum is generally estimated a priori by means of turbidity measurement and, as such, is subject to uncertainty. 

The ability of intracellular invasion of pathogenic species is commonly associated to virulence factors termed invasins. In *S. aureus*, invasins such as the fibronectin-binding proteins FnBPA and FnBPB are encoded by accessory genes that exhibit different allelic variants or can even be missing depending on the strain type [10]. This implies that the assessment of the heterogeneous cellular invasiveness within the same species could require varied testing conditions. Information on the diverse efficiency of cell invasiveness of the main strain types of major pathogens is largely missing. This lack of information relevant for the characterization of the strain types and for unveiling their underlying virulence strategies needs to be addressed by robust means of assessment. 

Currently, a parameter broadly used to express the invasiveness of a bacterial strain is the per cent of internalised bacteria (PIB) (Table 1). However, despite its frequent use, it is still unclear to what extent PIB values can be influenced when varying or adapting the MOI for the study. 

To obviate some of these shortcomings and improve the possibility of cross-comparison among *S. aureus* strains, the authors of a recent paper resolved to adopt a reference strain and normalise the number of internalised bacteria of the different isolates [11]. The invasiveness of each single strain was thus finally expressed as a percentage with respect to the internalization observed with a control reference strain, so reducing the level of inter-assays uncertainty. The normalization with a reference strains is certainly an important step forward to standardization. However, an ideal reproducible parameter that can be applied across different species with strain types exhibiting large differences of invasiveness is still far from being reached. 

The present study aims at exploring the relationship between inoculum size and extent of internalization. We could gain important insights on microbial cell invasion in osteoblasts from an in depth investigation of 22 clinical strains obtained from implant-related infections. The correlation between inoculum size and extent of internalization was analysed in detail and defined over a broad range of MOIs. New parameters to quantitatively express cell invasiveness that emerged from our findings were compared with the conventional ones currently in use. 

## 2. Materials and Methods

### 2.1. Bacterial Strains

The bacterial strains investigated included 8 *S. aureus*, 7 *S. epidermidis*, 5 *S. lugdunensis* and 2 *E. faecalis* clinical isolates from post-surgical orthopaedic infection and the *S. aureus* reference strain ATCC25923. All clinical isolates belong to the strain library of the Research Unit on Implant Infection, at the Rizzoli Orthopaedic Institute (Bologna, Italy). In addition to identification by classic phenotypic methods, all clinical strains were taxonomically confirmed and ribotyped by automated ribotyping using the Microbial Characterization System RiboPrinter (Qualicon DuPont, Willmington, DE, USA) as earlier described [12,13,14]. The similarity of the ribotype patterns generated by the RiboPrinter allowed strain subtyping into ribogroups. In addition to the identification of the ribogroup, *S. aureus* isolates were further processed for multi-locus sequence typing (MLST) and *spa* typing, which is based on the polymorphisms of the *Staphylococcus* protein A, as previously described by Montanaro et al. (2016) [14]. 

All staphylococcal and enterococcal isolates included in this study were sensitive to the gentamicin antibiotic as detected by phenotypic methods. *S. epidermidis* isolates were also tested for resistance to aminoglycoside antibiotics by genotypic methods as earlier described [15]. All isolates were from implant-related orthopaedic infections except for two strains, *S. epidermidis* cra1275 and *S. lugdunensis* cra1750, which were isolated from an orthopaedic infection non-associated to implant materials (see Table 2 and Table 3).

### 2.2. Preparation of the Bacterial Inoculum for Cell Invasion Assays

Bacterial isolates were thawed from frozen stocks of the strain library of the Research Unit on Implant Infections and plated on Tryptic Soy Agar (TSA, Biolife). For the cell invasion assays, bacteria were grown in Tryptose Broth (TB) (Biolife) at 37 °C for 18 h. Bacterial cultures were centrifuged at 4000 RCF for 15 min and the pellets obtained were resuspended in MEM growth medium (Sigma-Aldrich, St. Louis, MO, USA) supplemented with 10% foetal bovine serum (FBS, Invitrogen, Carlsbad, CA, USA) and 2 mM L-glutamine (Sigma-Aldrich). Bacterial concentration was estimated by optical density (OD) at 550 nm using a Hewlett Packard G1103A spectrophotometer. However, for the accurate assessment of the titre of viable bacteria, all starting bacterial suspensions were quantified in terms of CFU by agar plating. Only this a posteriori enumeration of CFU of the inoculum was used for studying the relationship between multiplicity of infection (MOI) and internalised CFU. Up to 17 serial 1:2 dilutions (last corresponding to a dilution factor of 1:65,536) were prepared starting from the initial suspension. For each single bacterial strain, up to 5 independent bacterial assays were performed and plotted together. 

### 2.3. MG63 Cell Culture

The human osteoblast-like cell line MG63 was purchased from ATCC (Rockville, MD, USA). MG63 cells were routinely cultured in MEM growth medium (Invitrogen), supplemented with 10% heat-inactivated foetal bovine serum (FBS, Invitrogen), 2 mM L-glutamine (Sigma-Aldrich) and penicillin/streptomycin (10,000 U/mL penicillin, 10 mg/mL streptomycin, Sigma-Aldrich) under standard culture conditions. MG63 cells were regularly subcultured three times a week. 

### 2.4. Invasion Assay of Osteoblasts in 96-Well Plates

**Figure 1:**
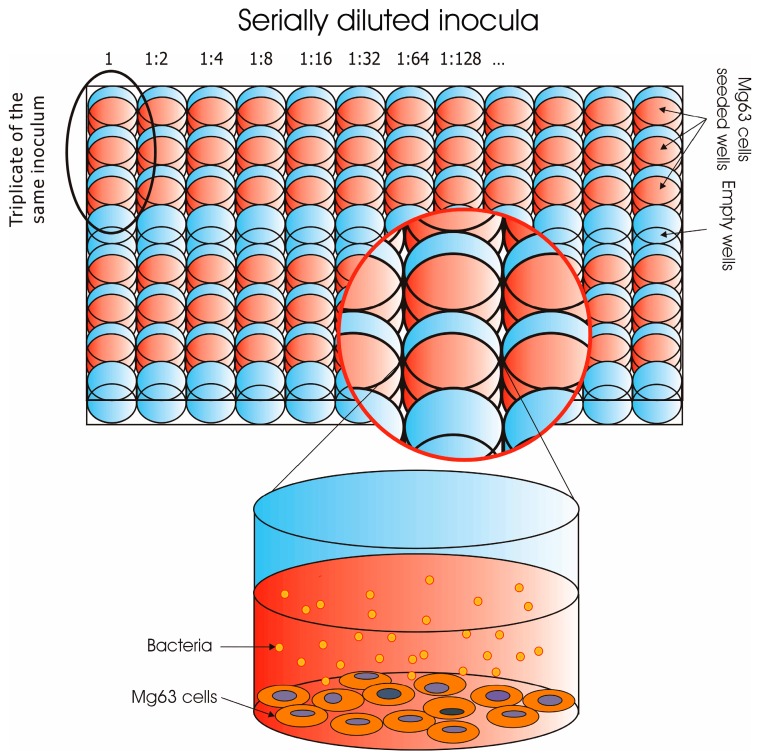
Scheme showing the organisation of the multiwell plates seeded with MG63 cells and subsequently treated with serial dilutions of bacterial suspensions. In the case of species incompetent for internalization, 10 dilutions of the starting bacterial suspensions were sufficient to reach levels with total absence of internalised bacteria and, thus, two bacterial strains could be contemporarily run on the same plate. Conversely, with *S. aureus*, greater dilutions needed to be reached.

The gentamicin protection assay was performed using 96-well tissue culture plates (Starlab Srl, Milano, Italy), slightly adapting the method earlier reported in Campoccia et al. (2016) [4]. MG63 cells were seeded at a cell concentration of about 5 × 10^3^ cells/well, in a volume of 100 μL of MEM growth medium without antibiotics. The seeded plates were incubated at 37 °C under standard cell culture conditions. After 24 h of culture, MG63 cells were found to closely approximate a density of 1 × 10^4^ cells/well [4]. Prior to exposing the osteoblast-like cells to the bacterial inoculum the wells of each plate were washed once with 200 μL of Dulbecco’s phosphate buffered saline (D-PBS, Sigma-Aldrich). One hundred microlitres of the initial bacterial suspension and its dilutions prepared as described above were added to triplicate wells, following the scheme illustrated in Figure 1. 

**Figure 2:**
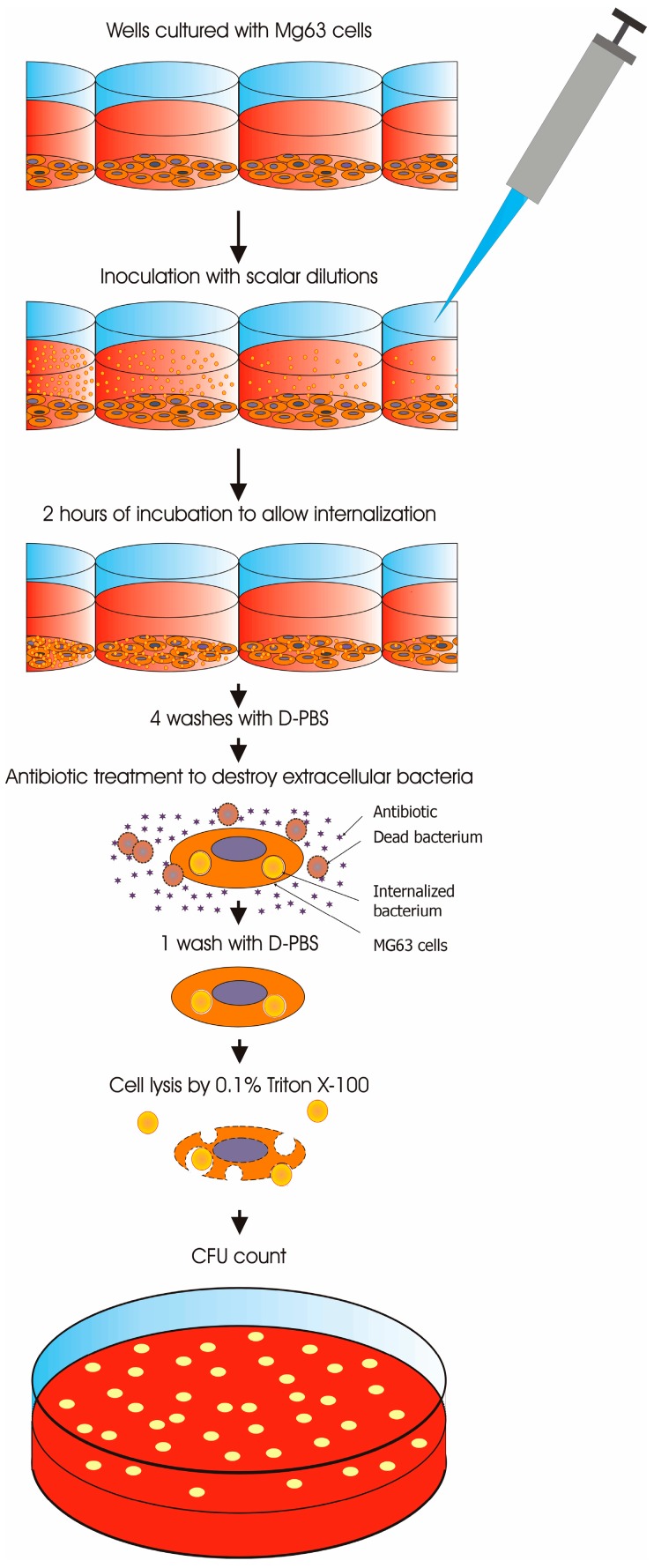
Gentamicin protection assay protocol utilised to assess bacterial internalization in MG63 cells.

After 2 h of incubation at 37 °C during which bacteria were allowed to invade the osteoblasts in culture, each well was washed 4 times with 200 μL of D-PBS and treated with MEM medium supplemented with either 100 or 200 μg/mL of gentamicin (Sigma-Aldrich) to kill all extracellular bacteria (a higher antibiotic concentration was used for *E. faecalis* on account of its intrinsic greater resistance) (Figure 2). At the end of the incubation, each well was washed once with 200 μL of D-PBS. The wells were then treated with 150 μL of 0.1% Triton X-100 for 5 min at 37 °C to lyse the eukaryotic cells and release intracellular bacteria. To quantify internalised bacteria, the cell lysates were processed for CFU count by plating 100 μL of bacterial suspension on TSA. 

The range of inoculum size finally considered was from about 2.5 × 10^2^:1 to 0.001:1 MOI (5 logs) for *S. aureus* and 10^4^:1 to 10:1 for less internalising species such as *S. lugdunensis*. Only MOI titres that gave a rate of internalization higher than 0 CFU were finally considered in the analyses. 

### 2.5. Statistics

Distribution plots, regression curves, curve equations and R-square values for the correlation of PIB vs. MOI, internalised CFU vs. Log_10_ MOI and Log_10_ CFU vs. Log_10_ MOI were achieved using the software Excel (Microsoft). Statistical comparison of the datasets by parametric (ANOVA followed by Bonferroni/Dunn test) and non-parametric (Kruskal–Wallis test followed by Kruskal–Wallis rank) tests was performed by StatView (version 5.0.1, Sas Institute Inc., Cary, NC, USA).

## 3. Results

### 3.1. PIB Values vs. Inoculum Size (MOI)

**Figure 3:**
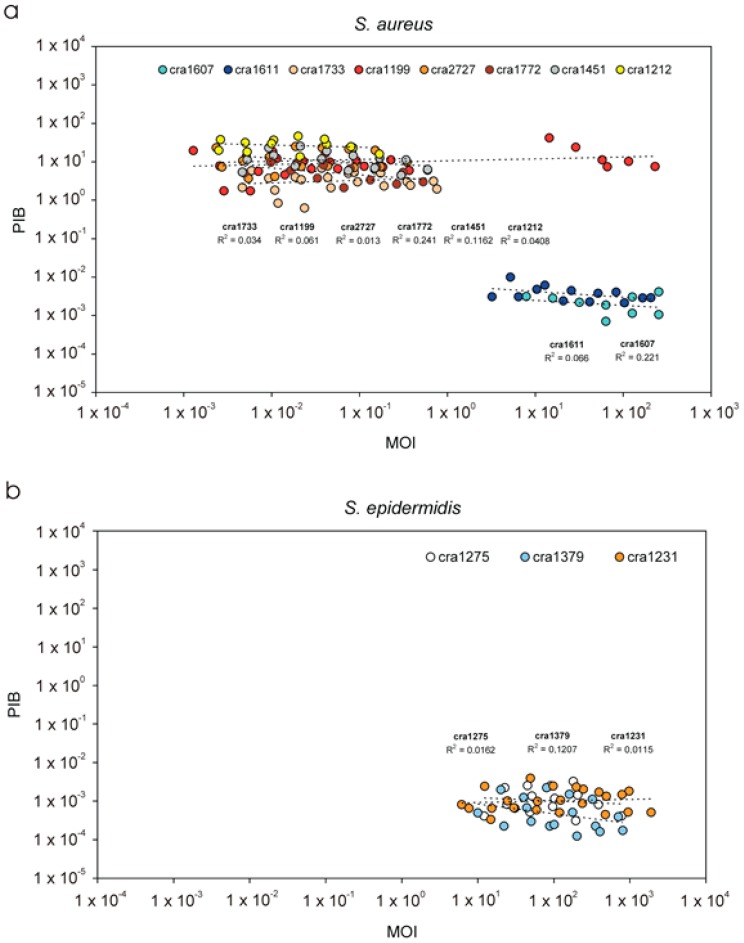
Variation of PIB (per cent of internalised bacteria) values obtained at different MOI (multiplicity of infection): (**a**) plot showing the distribution of each PIB value as a function of the inoculum size for all six *S. aureus* isolates; and (**b**) corresponding plot concerning three different *S. epidermidis* strains (those tested in three independent experiments). The R-square values of the PIB vs. MOI regression curve achieved for each single strain are reported in both graphs. It may be noticed as R-square values are generally very low and, in the broad range of MOI examined, any effect associated to the MOI appears negligible, especially in view of the large uncertainty of PIB measurements.

PIB, whose definition is reported in Table 4, is regularly used to express the degree of invasiveness of prokaryotic cells toward eukaryotic cells. Depending on the bacterial species and on the target eukaryotic cell line investigated, PIB is often obtained adopting different inoculum sizes. In the attempt to understand if and to what extent the MOI could influence PIB values, we examined the correlation existing between the two parameters. Figure 3 illustrates the plots obtained for different *S. aureus* and *S. epidermidis* strains. It may be noticed that, over a broad range of inoculum sizes, MOI did not appear to affect PIB values, as proven by the low R-square values for the regression curves calculated for the different strains. To avoid the possibility of drift when analysing a restricted number of PIB values, for *S. epidermidis* only the strains that were independently tested at least three times were considered. Overall, the uncertainty of measured PIB values appeared to weigh more than possible effects eventually associated to the size of the inoculum itself. Based on these finding, at least for the species investigated, PIB values can be used for comparison of strain invasiveness without fearing major effects derived from the varying MOI. 

Examining the data concerning the two staphylococcal species, Figure 3a shows that, although they belong to the same MLST clonal complex (CC30), *S. aureus* strains exhibited two distinct behaviours. Six strains, cra1733, cra2727, cra1772, cra1451 cra1199 and cra1212, were found highly invasive toward MG63 cells, while two isolates, cra1607 and cra1611, both belonging to the same t012 spa type, showed a very low level of intracellular invasiveness with PIB values up to three orders of magnitude lower, nearly matching *S. epidermidis* strains previously found incompetent to invade osteoblasts [4].

### 3.2. Internalised CFU vs. MOI Regression Curves

**Figure 4:**
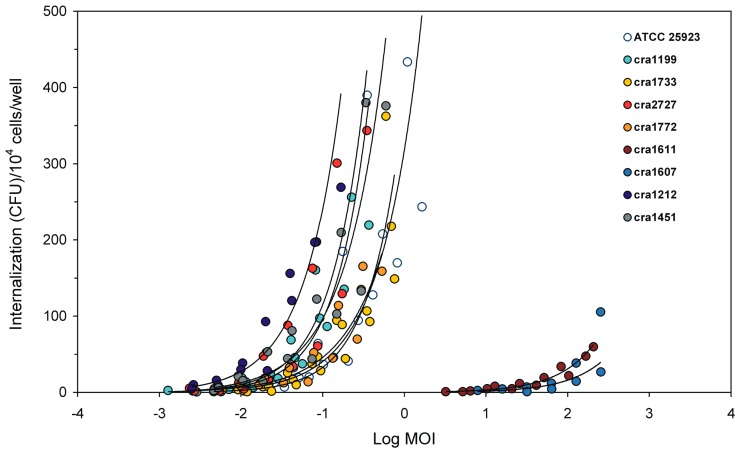
Correlation of CFU vs. MOI on semi-logarithmic scale. The regression curves of internalised CFU as a function of the 10-base logarithm of MOI are shown for all *S. aureus* strains.

**Figure 5:**
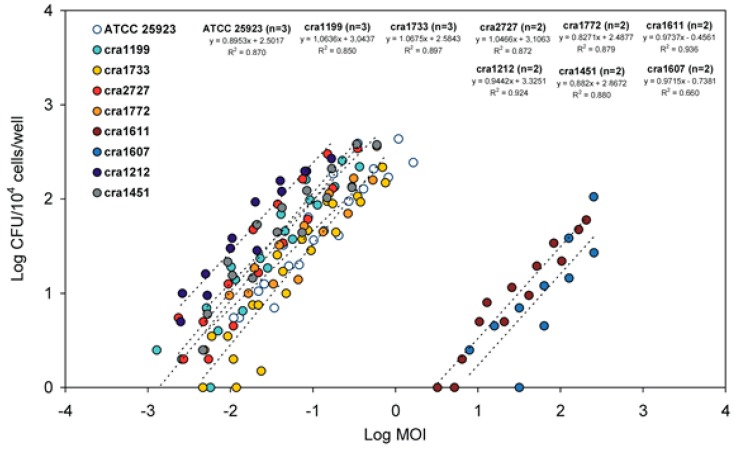
Correlation of internalised CFU vs. MOI. This dispersion plot based on a logarithmic scale shows the first-order linear regression curves obtained for each of the nine *S. aureus* strains. Per each strain, the number of experiments performed, the equation of the regression curve and the R-square value are reported. For *S. aureus* strain cra1199, only the three experiments performed on the low scale of MOI are plotted. When including the additional two experiments performed for cra1199 on the top of the scale of MOI, the R-square was found to increase from the value reported here of 0.85 to 0.97.

An in-depth investigation was performed to understand the type of relationship linking the inoculum size to the number of internalised bacteria. Early observations were conducted on semi-logarithmic graphs achieved by plotting the number of internalised CFU against the logarithm of MOI values (Log MOI). Figure 4 illustrates the examples of regression curves obtained for all *S. aureus* strains. Our attention however rapidly shifted to logarithmic curves, where both MOI and CFU were plotted in form of logarithm (Figure 5). In logarithmic scale, first-order linear regression curves achieved by dispersion plots were found to generally exhibit R-square values greater than 0.85 (over 70% of the strains). A good fit with R-square values higher than 0.95 was observed in three out of seven *S. epidermidis* strains. In the case of the *S. aureus* strain cra1199, the R-square reached a value of 0.97. For this strain, five independent assays, two of which conducted in the top range of MOI, were plotted together. Determination of internalised bacteria in the top range of MOI was normally avoided because requiring an overwhelming work for plating serial dilutions. Even so, of note, up to 51 agar plates were normally needed to assay a strain in a single experiment (17 MOI dilutions tested in triplicate). Overall, only very few strains exhibited R-square values lower than 0.70 (one out of nine *S. aureus* strains; one out of seven *S. epidermidis* strains; and one out of two *E. faecalis* strains). It is unclear what major factor could affect the goodness of fit of these few strains. Despite the replication of the experiments, the distribution of internalised bacteria appeared more irregularly scattered. Nonetheless, the regression curve remained similar to those found for the other strains of the same species (see for instance the dispersion plot of the cra1611 and cra1607 in Figure 5). 

### 3.3. New Parameters for Describing the Internalization Efficiency 

The discovery that, on a logarithmic scale, the distribution of internalised CFU is well approximated by first order linear regression curves opened a new scenario and the unexpected opportunity of extrapolating from the equations of these curves new parameters, suitable to qualify the level of invasiveness of bacterial strains. Among others, these were some of the parameters identified: the Internalization Minimal Inoculum (IMI), the Internalization at 1:1 MOI Inoculum (I1M) and the respective logarithmic values (LIMI and LI1M). A definition for all these parameters is provided in Table 4. All of them can simply be extrapolated from the equation of the first order regression curve, which is of the type:

Log_10_ (internalised CFU) = A × Log_10_ (MOI) + B (1)

where A and B values are the two constants of the linear equation. 

IMI was meant to be the inoculum value (in terms of MOI) at which 10^4^ eukaryotic cells internalise a single bacterium, i.e., the lowest concentration at which internalization occurs under the test conditions used. It has to be pointed out that this type of investigation requires the testing of numerous MOI titres in triplicate. This is feasible with our technique based on a 96-well microplate using 10^4^ eukaryotic cells per well, but it would be rather laborious with culture plates with a reduced number of wells (up to nine six-well plates would be required for testing a single strain). 

LIMI and IMI are extrapolated simply posing the number of internalised CFU = 1 and consequently Log_10_ (internalised CFU) = 0. Solving the equation, the IMI value is obtained as follows:

Log_10_ (internalised CFU) = 0 = A × Log_10_ (MOI) + B (2)

Log_10_ (MOI) = −B/A = Log_10_ IMI = LIMI (3)

IMI (MOI value with CFU = 1) = 10^(−B/A)(4)

The other two parameters that are even more promptly extrapolated from the equation are I1M and its logarithmic value (LI1M). I1M expresses the extent of internalization calculated for a hypothetic 1:1 MOI. At such a MOI value, corresponding to a bacterial suspension numerically equivalent to the eukaryotic cells in culture (in this case 10^4^ osteoblasts), Log_10_ (MOI) = 0. Once substituting the value into the equation:

Log_10_ (internalised CFU) = A × 0 + B = B (5)

Log_10_ (internalised CFU) = B = LI1M (6)

I1M = CFU (internalised bacteria with MOI = 1) = 10^B(7)

### 3.4. Invasiveness of S. aureus Strains

In addition to PIB analysis, all *S. aureus* isolates were processed for assessing the level of internalization into osteoblast cells as a function of the titre of the inoculum. After plotting the internalization data of each isolate in semi-logarithmic and logarithmic scales (Figure 4 and Figure 5), it became obvious even visually that *S. aureus* strains could be clustered into two distinct groups based on their ability of internalization: a large, major group of seven isolates (also including the reference strain ATCC 25923) characterised by a high invasiveness and another one, enlisting just two isolates (cra1611 and cra1607) with a remarkably different, very low ability of internalization. These findings are consistent with what illustrated in Figure 3a, when plotting PIB data as a function of MOI. 

Interestingly, both these strains belong to the same MLST clonal complex CC30 that includes all nine *S. aureus* strains here investigated (Table 2) [14]. Additionally, as reported in Table 2, cra1607 and cra1611 share the same spa type t012 with three other invasive strains, cra1199, cra1451 and cra1212. This heterogeneity among isolates belonging to the same strain type highlights that, giving the high variability in accessory genes, same strain type based on current bacterial typing criteria does not correspond to same bacterial behaviour. A similar conclusion was also reached when analysing the relationship between ribogroup or strain type vs. biofilm formation [16,17].

Table 5 reports in extenso all the measured parameters for expressing the level of invasiveness. It may be noticed that: (i) for the group of highly internalising (HI) *S. aureus* strains, up to 11% of the inoculum was taken up by osteoblasts in a 2 h interval versus just 0.0029% observed for the low internalising (LI) strains; (ii) examining the IMI values, internalization can be expected with a minimal inoculum as low as 14 CFU in HI strains against an inoculum of up to 4.3 × 10^4^ CFU for LI strains; (iii) I1M measures suggest that, with an inoculum of 10^4^ CFU (1:1 MOI) over 890 bacteria are taken up by osteoblasts in the case of HI strains against not even 1 for LI strains; (iv) LIMI and LI1M are parameters of less immediate interpretation, but both show a markedly decreased coefficient of variation with respect to PIB, IMI and I1M, thus resulting more robust parameters to be used as measures of invasiveness; and (v) LIMI is a logarithmic measure inversely proportional to the invasiveness of the strain and ranged from about −3 for the HI strains (with a C.V. of just 12% vs. 74% for PIB, 83% for IMI and 74% for I1M) to 0.6 for the LI strains (C.V. of 33% vs. 44% for PIB, 46% for IMI and 44% for I1M). LI1M assumes a value of 0 when with a 1:1 MOI inoculum a single bacterium is taken up by osteoblasts and of 4 when the entire inoculum is taken up. LI1M was of about 2.8 for HI strains and of −0.6 for LI strains (C.V. was similar to that found for LIMI). When the results concerning LIMI and LI1M were statistically analysed, the Bonferroni/Dunn test gave a *p*-value less than 0.0001 when comparing HI and LI strains.

**Figure 6:**
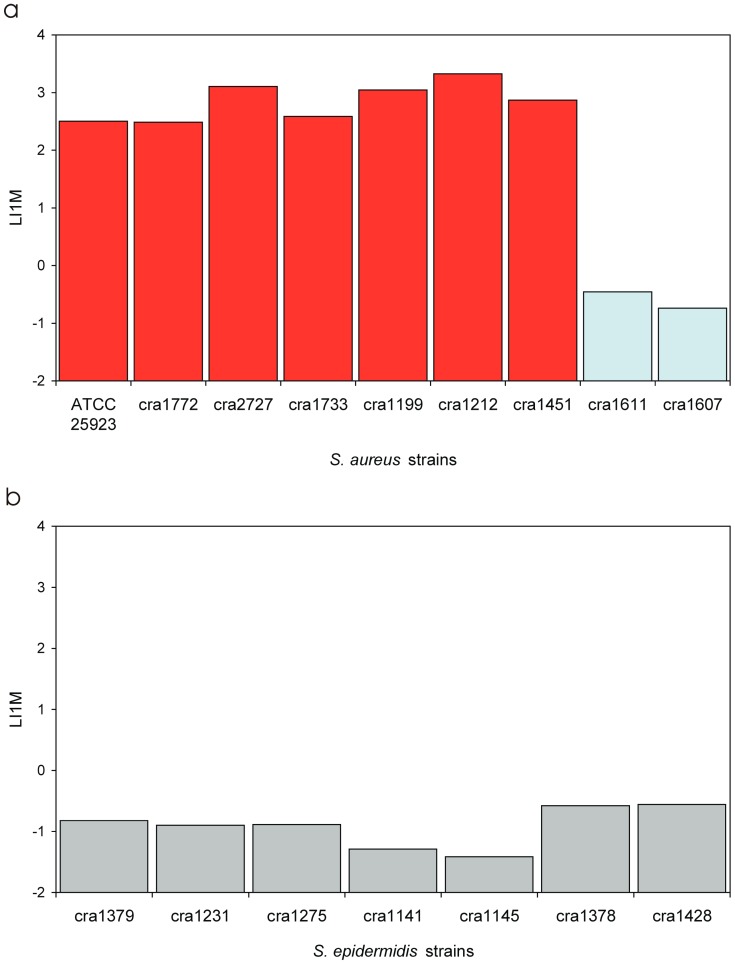
Bacterial invasiveness expressed in terms of LI1M: (**a**) LI1M value calculated for each *S. aureus* strain (HI *S. aureus* strains are in red, while LI strains are in light blue); and (**b**) LI1M value calculated for each of the seven *S. epidermids* strains.

**Figure 7:**
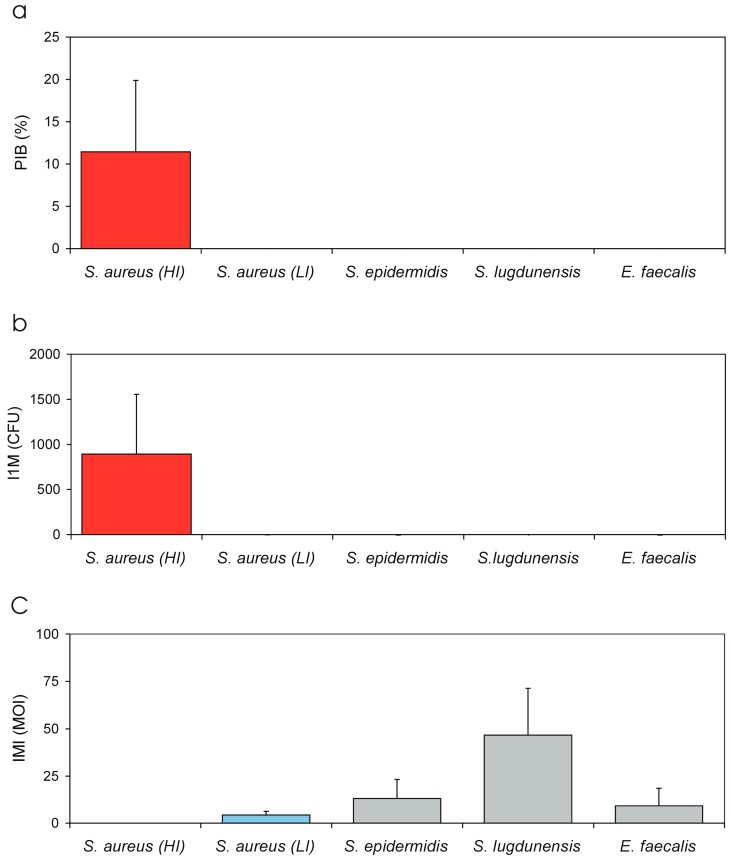
Different invasiveness across all investigated bacterial species expressed in terms of: (**a**) PIB; (**b**) I1M; and (**c**) IMI. Bars represent mean values ± S.D. Bar graphs with PIB and I1M values consistently show the gap between HI *S. aureus* strains and the LI strains and the strains of all other species. In the case of IMI, the higher is the internalization minimal inoculum. The lower is the invasiveness. Thus, IMI could also be interpreted as a measure of the resistance to phagocytosis. Here, even when compared to other incompetent species, *S. lugdunensis* exhibits the highest IMI and, therefore, emerges as the species with the lowest uptake by osteoblasts.

**Figure 8:**
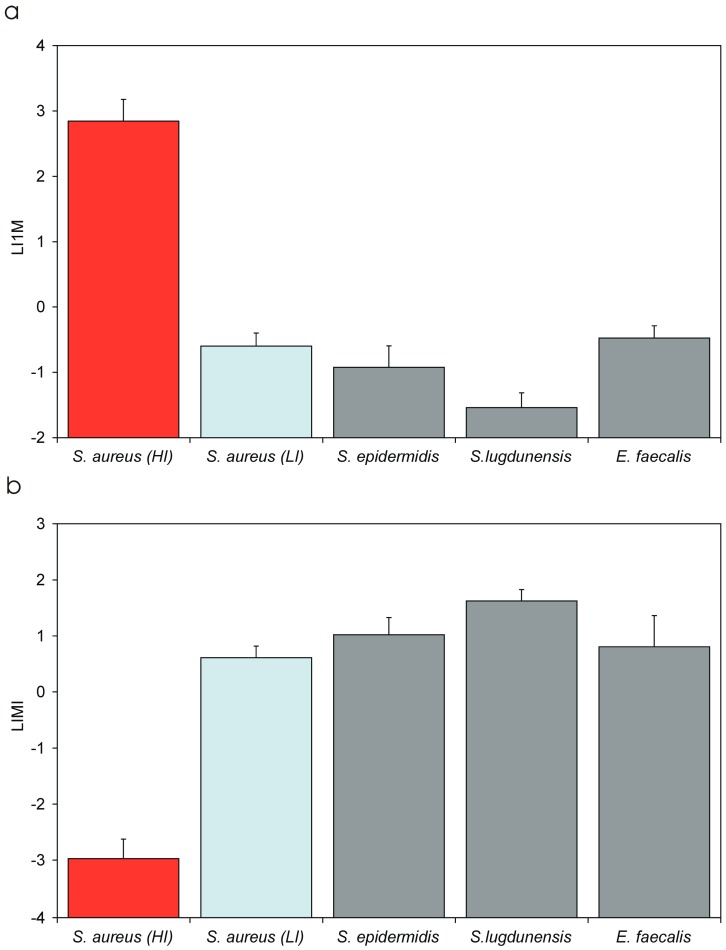
LI1M (**a**); and LIMI (**b**) values across the different microbial species investigated. Bars represent mean values ± S.D.

In the case of the most internalising strain, i.e., cra1212, with an IMI of 0.0003:1 MOI, as few as three bacteria were theoretically sufficient for internalization. This level of invasiveness is remarkable considering the time required by bacteria for sedimentation on the bottom of the well, where osteoblasts are adhered. The disabled internalization observed for cra1607 and cra1611 implies a shift greater than three orders of magnitude. It also emphasises the importance of mechanisms involved in the pathogenesis of internalization, implicating a marked heterogeneity and a huge jump in invasiveness when examining *S. aureus* strains belonging to the clonal complex. These differences can be well appreciated also in Figure 6a, which reports the L1IM values of all *S. aureus* isolates, and in Figure 7, where PIB, I1M and IMI values of *S. aureus* HI and LI strains and of the other investigated bacterial species are compared. The use of LI1M and LIMI appear convenient for their easy extrapolation from the equation and enable to highlight bacterial strains/species exhibiting respectively high and low internalization levels (Figure 8a,b). Conversely, PIB as well as I1M (Figure 7) promptly distinguish effective internalising bacterial strains with respect to poorly internalising ones, which appear flattened down. Interestingly, inversely related to invasiveness, IMI emerges an ideal parameter to measure the resistance to phagocytosis and could probably be used in studies aimed at interfering with internalization, responding to the basic question of how large an inoculum has to be for the internalization to occur. 

### 3.5. Invasiveness of S. epidermidis Strains

**Figure 9:**
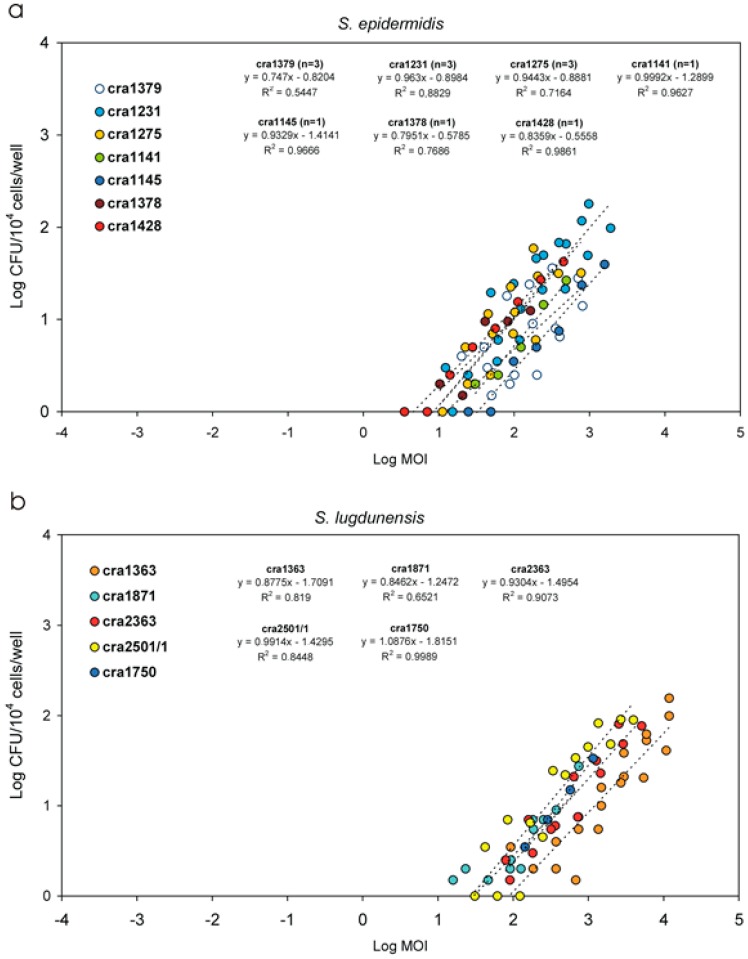
Log CFU vs. Log MOI linear regression curves: *S. epidermidis* strains (**a**); and *S. lugdunensis* strains (**b**).

The Log CFU vs. Log MOI linear regression curves observed for the *S. epidermidis* isolates are reported in Figure 9a. Except for one isolate, the R-square values found for the different isolates were all greater than 0.70 and for nearly half of the isolates the R-square value was greater than 0.96. First order linear curves appeared to run nearly parallel, with a slope similar to those found for the *S. aureus* strains, the lowest invasiveness being associated simply to a shift of the line to the right part of the graph. 

The seven *S. epidermidis* clinical isolates investigated belong to six of the most frequent ribogroups among orthopaedic infections. All of them demonstrated very low levels of invasiveness. Thus, within this species, the rates of internalization appeared homogeneous. When analysing the invasiveness across all bacterial species, differences emerged with the highly invasive HI *S. aureus* strains, but also with *S. lugdunensis*, which was the species exhibiting the lowest rate of internalization in absolute. 

With LIMI as well as of LI1M, the logarithmic normalization enabled the versatile use of ANOVA test in combination with a post-ok test such as the Bonferroni/Dunn test. Significant differences in LIMI and LI1M values were observed when comparing *S. epidermidis* strains with HI *S. aureus* strains (LI1M and LIMI, *p* < 0.0001) and *S. lugdunensis* (LI1M, *p* = 0.002; LIMI = 0.004). Conversely, the invasiveness of *S. epidermidis* was found to be similar to that of the LI *S. aureus* strains (LI1M, *p* = 0.189; LIMI, *p* = 0.122) and of *E. faecalis* (LI1M, *p* = 0.076; LIMI, *p* = 0.404). As illustrated in Table 5 and Figure 6b and Figure 8, *S. epidermidis* LI1M values greatly diverged from those of HI *S. aureus* strains (−0.92 ± 0.33 vs. 2.85 ± 0.33), were only slightly different from LI *S. aureus strains* (−0.60 ± 0.20) and *E. faecalis* (−0.47 ± 0.19), and were higher than *S. lugdunensis* (−1.54 ± 0.23). 

### 3.6. Invasiveness of S. lugdunensis Strains

**Figure 10:**
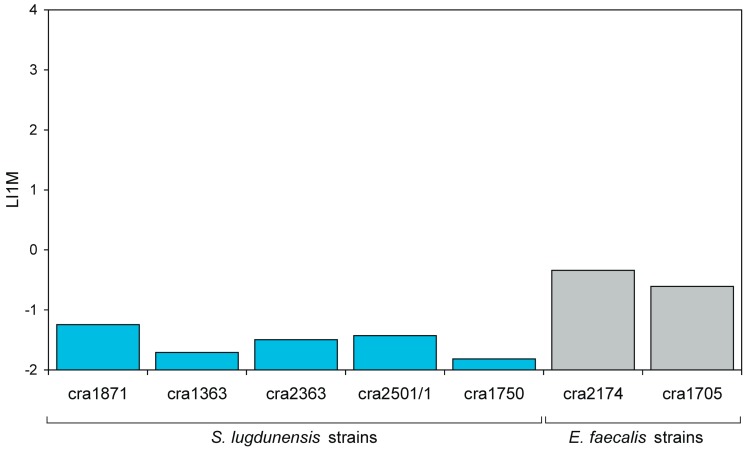
LI1M values calculated for *S. lugdunensis* and *E. faecalis* strains.

*S. lugdunensis* was by far the least intracellularly invasive species (Table 5 and Figure 7c and Figure 8). In Figure 9b, the regression curves obtained for the five strains investigated are reported. Again, the curves of the strains appeared to run approximately parallel. This was confirmed also by statistical analysis. The means of the slopes of the curves obtained for the three staphylococcal species exhibited only slight variations and were found not to differ significantly when analysed by ANOVA followed by post-ok test. After non-parametric analysis and Kruskal–Wallis ranking, *S. lugdunensis* strains exhibited the lowest PIB and I1M mean values and, accordingly, the highest MOI necessary for a single internalization event to occur (see Figure 8c). Figure 10 shows the LI1M value that was extrapolated per each strain. LI1M mean value for *S. lugdunensis* differed from that of all other species investigated, qualifying this species as the less intracellularly invasive among the investigated. 

### 3.7. Invasiveness of E. faecalis Strains

Just two clinical isolates of *E. faecalis* were considered in this study, which was primarily aimed at exploring new ways to quantify bacterial efficiency of internalization. The two *E. faecalis* strains appear to follow the same trend of LI *S. aureus* and *S. epidermidis* strains. While Figure 7a,b promptly help to identify *E. faecalis* amongst the least osteoblast invasive species, Figure 7c and Figure 8 support in finely resolving the ranking of the strains of these less invasive species. 

## 4. Discussion

Presently, the most used parameters to express the intracellular invasiveness of a bacterial strain are the number of internalised bacteria (IB) and PIB, both of which are described in Table 1. However, there is limited information about the influence of MOI used for the tests on both these parameters. In past literature, it has been assumed that, at highest MOI, a plateau could be reached, where NIB did not increase any further. In view of this, the MOI was sometimes chosen in proximity to what was considered a plateau, thinking about the advantage in reducing the variability in the NIB value measured [5,7]. Nonetheless, this starting hypothesis has important implications. For instance, assuming the possibility of a different behaviour among strains, per each bacterial strain investigated, a preliminary study should be done to check for the plateau before testing either for NIB or PIB. 

Moreover, given the remarkable variations in cell invasiveness among bacterial species, tests are frequently performed using different target MOI. On the one hand, this approach facilitates NIB assessment enabling high CFU counts even in the case of less invasive strains, but, on the other hand, the possibility of comparison could be compromised should parameters such as NIB and PIB be affected by MOI variations. Here, in addition to investigating the relationship between internalised CFU, PIB and MOI in detail, we looked at new parameters that could overcome some of the current limitations. Unexpectedly, alternative possibilities to measure bacterial invasiveness were actually generated by the discovery of a first order linear regression linking the NIB value to the inoculum size over a broad range of MOI. 

As far as this relationship is concerned, with some difficulties we pursued the objective of searching a posteriori to find out if somebody else had already observed it in the past. The vast majority of the investigations in the literature were found to address the relationship between internalization and time of exposure. However, we discovered that, as early as 1994, while studying the invasion of rabbit ileal tissue by *Enterobacter cloacae*, de Kort et al. reported the existence of a linear regression with a high correlation coefficient (R-square: 0.90) between the inoculum and gentamicin resistant bacteria, both expressed as Log CFU [18]. This finding was reported from the experimental work conducted with a different organotypic culture model, but was already suggesting the rule possibly governing the relationship between internalization and inoculum. 

More recently, Donnarumma et al. (2004) introduced a figure illustrating the internalization of *S. aureus* as a function of MOI in human keratinocyte (HaCat) cells, pre-incubated or not pre-incubated with α-melanocyte stimulating hormone (α-MSH) [19]. The authors did not use the logarithm of MOI, but they reported the MOI value in a sort of exponential scale (three categories of 1, 10 and 100 MOI, respectively). Interestingly, for untreated as well as α-MSH treated keratinocytes, the relationship Log CFU vs. MOI (in exponential scale) appears to approximate two distinct first order linear regression curves, with treated cells showing lower levels of internalization. The authors did not comment or further investigate these easily identifiable linear curves of the plot and all of their implications. Focussed on the important aim of the study, they reported that, by down-regulating the keratinocytes expression of integrins such as β1 and HSP 70, both capable of interacting with *S. aureus* FnBPA/B invasins, α-MSH causes a reduced internalization of bacteria into host cell. A posteriori, we believe that the study of the impact of inhibitors of internalization such as α-MSH on the equations of the Log CFU vs. Log MOI regression curves would have been of extreme interest. 

Our observations confirm the validity of PIB, demonstrating that, at least for the system bacteria/osteoblasts (but very likely also with any other eukaryotic cell type, given the evidence highlighted above from the previous studies with ilieal organotypic cultures and keratinocytes), this parameter can be used over a large interval of MOI without fearing the influence of the inoculum size. However, even within the same bacterial strain, PIB measurement is affected by considerable uncertainty (Figure 3). For instance, the PIB for ATCC 25923 *S. aureus* reference strains was of 4.5 ± 2.6 (thus exhibited a C.V. greater than 58%). It has to be noted that, in this study, the definitive PIB value attributed to a bacterial strain was not calculated from a single measurement in duplicate or triplicate as it is normally done, but it was rather obtained per each strain by averaging all PIB values obtained at different MOI in triplicate and in different experiments. This means that, with conventional single measurements of PIB, the level of uncertainty could be expected to be even greater. The same consideration applies to the PIB results expressed in Table 5, which reports the mean PIB value for different strains of the same species. Moreover, PIB values do not follow a normal distribution and require the use of non-parametric statistics or, otherwise, should be processed for logarithm normalization. 

Apart from the above limitations, our observations validate the use PIB values. Conversely, NIB values are strongly depending on MOI. Thus, NIB can be used for cross-comparisons only with bacteria tested with exactly the same inoculum. Just within these strict circumstances, NIB could therefore be used for expressing the internalization as a per cent with respect to a reference strain. This latter approach would increase the uncertainty, as there would be a contribution of additional uncertainty derived also from the measurement of NIB for the reference strain. However, in return, the use of a reference strain can certainly improve inter-laboratory reproducibility.

The two extrapolated parameters IMI and I1M provide a different type of information, the former expressing how high the MOI of a strain should be for the internalization to occur and the latter the extent of internalization at a 1:1 MOI. IMI can be used to rank strains based on their lowest degree of internalization or, seen from another point of view, their highest resistance to internalization/phagocytosis. In our past study [4], we were actually unable to compare different species that were tested with different inoculum except when comparing highly internalising *S. aureus* with species appearing incompetent to invade osteoblasts. As visible in Figure 7c, IMI facilitates the task of comparing less invasive species and *S. lugdunensis* promptly emerges as the less invasive species. On the contrary, I1M somehow parallels PIB. Its value was found to range between 0.03 and 892 CFU for the different species investigated, with respect to a range varying from 0.0003 to 11.4% for PIB. The C.V. of the measures performed was similar for PIB as for I1M, indicating similar uncertainty of the measurements. In view of the proven lack of variation in the range of MOI considered, PIB certainly remains a parameter less laborious to measure than I1M. On the other hand, I1M is obtained from a curve with more data points and can be thought less sensitive to drifts. PIB, IMI and I1M are anyway affected by a non-normal distribution with broadly scattered data, requiring non parametric analysis. I1M could find a convenient use when screening the different potential invasion of a strain with different eukaryotic cell types.

LIMI and LI1M, the logarithmic transformation of IMI and I1M, appear versatile and of easy use. Now that a first order linear relationship has been proven, the need for extensive data obtained at different MOI is reduced and so is the need for numerous 1:2 dilutions of the inoculum. Figure 8 summarises the LIMI and LI1M results on Table 5. As described for IMI, even LIMI can be thought as a parameter pinpointing lack of invasiveness of a strain and, thus, it can be conveniently applied in tests with substances interfering with internalization. Both LI1M and LIMI exhibit a more reduced C.V. than non-logarithmic transformed measurements and, thus, result more robust parameters to assess the efficiency of internalization. 

The investigation of how and to what extent internalization inhibiting drugs can impact the Log CFU vs. Log MOI regression curves by affecting either their slope, by causing a shift of the curve or affecting the first order linearity, depending on their specific mechanism of action, warrants further research work. Equally important could be the analysis of the effects in terms of IMI or LIMI when investigating the activity of factors interfering with bacterial phagocytosis or internalization. 

Overall, this study reconfirms the incompetence of orthopaedic clinical isolates of *E. faecalis* as well as of coagulase negative species such as *S. epidermidis* and *S. lugdunensis* to invade osteoblasts. The new investigation enabled us to reveal three distinct degrees of intracellular invasiveness across the strains and the species investigated, as substantiated by statistical analysis of LI1M values. *S. aureus* strains were prevalently highly invasive of osteoblasts (LI1M: 2.85 ± 0.33) except for two strains (LI1M: −0.60 ± 0.20), which were found positive to both FnBPA/B invasins (unpublished data), but seemingly lacking a functional allelic form. LI *S. aureus* strains were found not to differ significantly from *S. epidermidis* and *E. faecalis*. A result that did not emerge from the past analyses is that *S. lugdunensis* exhibited a distinguishable, even lower, degree of internalization, statistically differing from LI *S. aureus*, *S. epidermidis* and *E. faecalis*. Probably for the restricted number of *E. faecalis* isolates investigated, the difference of LIMI with respect to *S. lugdunensis* was insufficient for statistical significance. It remains to be seen the reason for the distinct behaviour of *S. lugdunensis* when compared to other species already earlier classified as incompetent to invade osteoblasts. Based on the IMI value found for this species, over 46 viable bacteria per each of 10^4^ osteoblasts are needed for a single event of internalization to occur. This finding suggests an impressive lack of interaction of this bacterial species with osteoblasts. 

Another important point of this work is that *S. aureus* strains belonging to CC30, no matter the type of the measurement, exhibit a bimodal distribution. Based on current findings, we actually question whether, for species such as *S. aureus*, invasiveness should be expressed by a mean value or each strain should rather be analysed and classified by identifying distinct levels of internalization. The parameters here proposed enabled us to discern two distinct clusters of *S. aureus* strains. More in general, for all microbial species, it would be important to verify the different degree of invasiveness determined by the various forms and assets of invasins, trying to relate phenotypic properties with genotypic virulence traits. Probably, for *S. aureus* as for other pathogens, multimodal behaviours can be expected. Therefore, it would be more informative and appropriate to describe the distribution across the microbial population based on defined thresholds of invasiveness. In other words, the mean PIB (8.89%) or the mean LI1M values (2.08) suggestive of a generally high ability of internalization are not well and punctually descriptive of our collection of CC30 *S. aureus* strains, which actually consists for 22% of LI strains comparable to *S. epidermidis* (PIB = 0.0029%; LI1M = −0.60) and only for 78% of highly invasive strains (PIB = 11.4%; LI1M = 2.85).

Under the static conditions used in conventional gentamicin protection assays, bacterial uptake by eukaryotic cells can be thought to be influenced by several factors: (i) bacterial sedimentation rate (influenced by the size, the shape and the tendency to agglomerate of the microbe as well as by the viscosity of the medium); (ii) the physiological composition of the culture medium (for instance the presence of host adhesins such as fibronectin); (iii) the type of cell line used in the assay (in particular its histological origin, its phagocytic activity—professional or non-professional phagocytic cells, in primary or secondary culture, level of expression of integrins capable of interaction with adhesins); and (iv) the bacterial strain type (the bacterial species, the strain specific endowment of genes encoding invasins and their allelic variants, the panel of adhesins mediating the adhesiveness to the surface of eukaryotic cells and so on). It may be possible to speculate that, under the experimental model used, *S. lugdunensis* not only lacks specific invasins, but also it poorly interacts with the surface of osteoblastic cells, adhering less than the other species investigated. Indeed, eukaryotic cells can actively ingest even inanimate microparticles of solid materials and, in the absence of specific mechanisms triggering active internalization, the level of internalization could be uniquely driven by the number of bacteria adhered to the cell surface. Conversely, the behaviour of *S. aureus* strains would seem characterised by a quantic jump, with strains either actively invading osteoblasts or, on the contrary, behaving like the cells of other bacterial species, possibly adhering on the surface of osteoblasts, but lacking active mechanisms of internalization. One can therefore speculate that the possession of invasins determines drastic changes in bacterial invasiveness between bacterial strains, while other properties such as the level of adhesiveness could further modulate and influence bacterial behaviour, probably determining subtle changes even among strains of species lacking invasins. 

## 5. Conclusions

We expect that our present findings will be the base for future studies aiming at characterising and comparing the internalization properties of the different strain lineages of cell invasive pathogens towards relevant eukaryotic cell types. The same parameters could also express a potential in studies aimed at comparing the susceptibility of different tissue cell types towards bacterial invasion. 

## Figures and Tables

**Table 1:** Current parameters in use for expressing the potential of invasiveness of bacterial strains.

Acronym	Description	Meaning
NIB	Number of internalised bacteria	Number of internalised bacteria (at a given MOI). Usually expressed in terms of CFU per well or CFU per number of eukaryotic cells, NIB is easily obtained, but its use presents limitations. Its value is influenced by the MOI in use. The assessment of internalization of bacteria belonging to different species is usually performed at different MOI, thus direct comparison of NIB among strains is hampered. Obstacles are met when strains of the same species exhibit remarkable differences in invasiveness, ideally needing different inoculum MOI.
PIB	Per cent of internalised bacteria	PIB value represents the per cent fraction of the inoculum that is taken up by the eukaryotic cells. Its value is directly proportional to the invasiveness of the test strain towards a specific eukaryotic cell type. Based on our current results, PIB is not significantly affected by MOI and is more appropriate than NIB for strains comparison.

**Table 2:** Characteristics of *S. aureus* strains.

Species	Strain	Ribogroup	MLST CC	*spa* Type	*spa* CC	Origin
*S. aureus*	ATCC 25923	-	CC30 *	t021	CC021/012	Clinical
	cra1733	cra-119-S-8				K
	cra2727					H
	cra1772			t298		IF
	cra1199			t012		EF
	cra1451	cra-138-S-2				H
	cra1611					IF
	cra1607					H
	cra1212	cra-53-S-7				EF

Legend: knee arthroplasty (K); hip arthroplasty (H); internal fixation systems (IF); external fixation systems (EF). * Within the MLST CC30, all isolates belonged to ST30 except for cra1772, which was associated to ST2954. The detailed characterization of these strains has earlier been reported in Montanaro et al. (2016) [14].

**Table 3:** Characteristics of *S. epidermidis*, *S. lugdunensis* and *E. faecalis* strains.

Species	Strain	Ribogroup	Origin
*S. epidermidis*	cra1379	cra-63-S-7	FI
	cra1231		H
	cra1275	cra-63-S-4	PSI
	cra1141	cra-92-S-5	H
	cra1145	cra-122-S-2	H
	cra1378	cra-119-S-4	H
	cra1428	cra-80-S-1	K
*S. lugdunensis*	cra1871	cra-62-S-1	FI
	cra1363		FI
	cra2363	cra-64-S-8	FI
	cra2501/1		FE
	cra1750	cra-74-S-5	No MD
	cra1871	cra-62-S-1	FI
*E. faecalis*	cra2174	cra-116-S-1	H
	cra1705	cra-115-S-8	FI

Legend: post-surgical infection (PSI); knee arthroplasty (K); hip arthroplasty (H); internal fixation systems (IF); no medical device (No MD). The prevalence among orthopaedic implant infections of the different ribogroups of *S. epidermidis* and *E. faecalis* have earlier been reported respectively in Campoccia et al. (2009) [12] and in Arciola et al. (2007) [13].

**Table 4:** Newly proposed parameters for expressing the invasiveness of bacterial strains.

Acronym	Description	Meaning
IMI	Internalization minimal inoculum	IMI is a virtual value extrapolated from the equation of the regression curve achieved by plotting Log MOI vs. Log (CFU). IMI corresponds to the lowest MOI required for the internalization of a single bacterium. The lower the value the higher the invasiveness of the strain. IMI lowest value, in our system, would correspond to 0.0001:1 MOI, i.e., a suspension containing a single bacterium for 10^4^ eukaryotic cells. Being inversely related to invasiveness, IMI could advantageously be used to express the resistance to phagocytosis.
I1M	Internalization at 1:1 MOI inoculum	I1M is a virtual value, extrapolated from the equation of the regression curve achieved by plotting Log MOI vs. Log (CFU). I1M corresponds to the number of bacteria internalised when hypothetically exposing each eukaryotic cell to a single bacterium (i.e., when using a 1:1 MOI). Its value is directly proportional to the degree of invasiveness of the strain.
LIMI	Log_10_ of the IMI value	LIMI is promptly obtained from the regression curve of Log MOI vs. Log (CFU). Its value, expected in the range from −3 to 4, is inversely proportional to the degree of invasiveness of the bacterial strain. LIMI exhibits a lower coefficient of variation with respect to IMI, PIB and I1M, and it more closely approaches a normal distribution. LIMI can be easily transformed to obtain the corresponding IMI value.
LI1M	Log_10_ of the I1M value	LI1M is promptly obtained from the regression curve of Log MOI vs. Log (CFU). Its value, expected in the range from −4 to 3, is directly proportional to the degree of invasiveness of the bacterial strain. LI1M exhibits a lower coefficient of variation with respect to IMI, PIB and I1M, and it more closely approaches a normal distribution. It can be easily transformed to obtain the corresponding I1M value.

**Table 5:** Invasiveness of the different bacterial species through old (PIB) and new parameters.

Bacterium	PIB [Mean ± S.D. ^1^/(C.V.)]	IMI [Mean ± S.D./(C.V.)]	I1M [Mean ± S.D./(C.V.)]	LIMI [Mean ± S.D./(C.V.)]	LI1M [Mean ± S.D./(C.V.)]
*S. aureus* (HI + LI)	8.89 ± 8.88 (99.9%)	0.97 ± 2.04 (211.1%)	693.67 ± 696.23 (100.4)	−2.18 ± 1.61 (74.1%)	2.08 ± 1.15 (74.3%)
*S. aureus* (HI)	11.43 ± 8.45 ^h^ (73.9%)	0.0014 ± 0.0012 ^l^ (83.2%)	891.79 ± 663.52 ^h^ (74.4%)	−2.97 ± 0.35 ^a,b,c,d^ (11.7%)	2.85 ± 0.33 ^a,b,c,d^ (11.6%)
*S. aureus* (LI)	0.0029 ± 0.0016 (43.9%)	4.34 ± 1.99 (45.7%)	0.27 ± 0.12 (44.4%)	0.61 ± 0.21 ^a,f^ (33.5%)	−0.60 ± 0.20 ^a,f^ (33.4%)
*S. epidermidis*	0.0010 ± 0.0005 (49.9%)	13.2 ± 10.0 (76.1%)	0.15 ± 0.09 (63.0%)	1.02 ± 0.30 ^b,e^ (29.6%)	−0.92 ± 0.33 ^b,e^ (35.5%)
*S. lugdunensis*	0.00027 ± 0.00012 ^l^ (46.2%)	46.6 ± 24.7 ^h^ (53.0%)	0.032 ± 0.016 ^l^ (50.8%)	1.63 ± 0.20 ^c,e,f^ (12.4%)	−1.54 ± 0.23 ^c,e,f,g^ (14.7)
*E. faecalis*	0.0011 ± 0.0011 (95%)	9.2 ± 9.37 (101.5%)	0.35 ± 0.15 (42.1%)	0.81 ± 0.55 ^d^ (68.6%)	−0.47 ± 0.19 ^d,g^ (39.8%)

S.D., standard deviation; C.V., coefficient of variation. ^1^ Note that the mean of PIB and the respective S.D. values were calculated after averaging the many measurements performed per each strain at different MOI. S.D. and C.V. are therefore lower than what could be found with conventional single measurements that are based just on a MOI. The non-parametric Kruskal–Wallis test followed by Kruskal–Wallis ranking was used to analyse the means differences for PIB, IMI and I1M. The tied-*p* values for the three comparative analyses were respectively of 0.0009, 0.0009 and 0.0008. HI *S. aureus* strains diverged from the behaviour of the strains of the other species previously described as incompetent. Here, only the more detailed statistical analyses considering *S. aureus* split into the two HI and LI subcategories are shown (HI, highly internalising strains; LI, low internalising strains). The heterogeneous behaviour of *S. aureus* strains appeared distributed in two distinct clusters clearly causing a bimodal distribution. Logarithmic normalization enabled the use of post-hoc cell analysis by the Bonferroni/Dunn test. For LIMI and LI1M, superscript letters within the same column indicate the cross-comparisons statistically significant after performing the test.
